# Supplementary material for: Soluble CD163 as a Marker of Macrophage Activity in Newly Diagnosed Patients with Multiple Sclerosis
Source: PLoS One. 2014 Jun 2;9(6):e98588. doi: 10.1371/journal.pone.0098588 (PMC4041861; doi:10.1371/journal.pone.0098588)
Supplement: Data S1 — File contains: Table S1 Table S1 includes Excel file and (double click to activate) contains all basic data for this paper. Abbreviations: RRMS (relapsing-remitting MS), PPMS (primary-progressive MS), SPMS (secondary-progressive MS), CIS (clinically isolated syndrome), SC (symptomatic controls with normal or abnormal MRI), n (number of persons), CSF (cerebrospinal fluid), y (years), d (days), Gender (1 = male; 2 = female), OND (other neurological disease), OMD (other medical disease). Table S2 Table S2 lists proteins, their respective molecular weight and ratio CSF/serum. The table is adapted from Nockher et al (22). Abbreviations: kDA (kilo Dalton). Table S3 Table S3 shows the output of our regression analysis on log transformed sCD163 serum values (lSerum) Table S4 Table S4 shows the output of our regression analysis on log transformed sCD163 CSF values (lcsv). Table S5 Table S5 shows the output of our regression analysis on log transformed scd163 CSF/serum values (lRatio). Table S6 Table S6 shows the output of our regression analysis on log transformed sCD163 index values (lIndex) Figure S1 Figure S1 shows the istribution of proteins and their CSF/serum ratios plotted against their molecular weight (in kD). This figure is adapted from Nockher et al (22). Proteins marked with (•) originate solely from the blood compartment (see appendix table 2 below) and proteins marked by (o) are suspected to be produced intrathecally. (DOCX) [file pone.0098588.s001.docx]

**Data S1**

**Table S1.**

Excel file below (double click to activate) contains all basic data for this paper. Abbreviations: RRMS (relapsing-remitting MS), PPMS (primary-progressive MS), SPMS (secondary-progressive MS), CIS (clinically isolated syndrome), SC (symptomatic controls with normal or abnormal MRI), n (number of persons), CSF (cerebrospinal fluid), y (years), d (days), Gender (1=male; 2=female), OND (other neurological disease), OMD (other medical disease).

| **Tabel S2: Proteins and their molecular weight** | | |
| --- | --- | --- |
| **Characteristic** | **Molecular weight (kDa)** | **Ratio CSF/serum'10E-3** |
| Molecule |  |  |
| Apolipoprotein E | 33 | 60 |
| Orosomucoid | 40 | 5.3 |
| alfa1 antichymotrypsin | 43 | 4.4 |
| C-reaktivt protein | 50 | 3.2 |
| soluble CD14 | 52 | 50 |
| alfa1 antitrypsin | 54 | 4.3 |
| Hemopexin | 57 | 3.4 |
| Transthyretin | 60 | 70 |
| Albumin | 66 | 4 |
| Transferrin | 81 | 7 |
| Plasminogen | 90 | 1.6 |
| soluble CD163 | 130 | 20 |
| Coeruloplasmin | 151 | 2.3 |
| IgA | 160 | 0.7 |
| IgG | 160 | 1.9 |
| Fibrinogen | 340 | 0.22 |
| Ferritin | 480 | 10 |
| Table S2 lists proteins, their respective molecular weight and ratio CSF/serum. The table is adapted from Nockher et al (22). Abbreviations: kDA (kilo Dalton) | | |


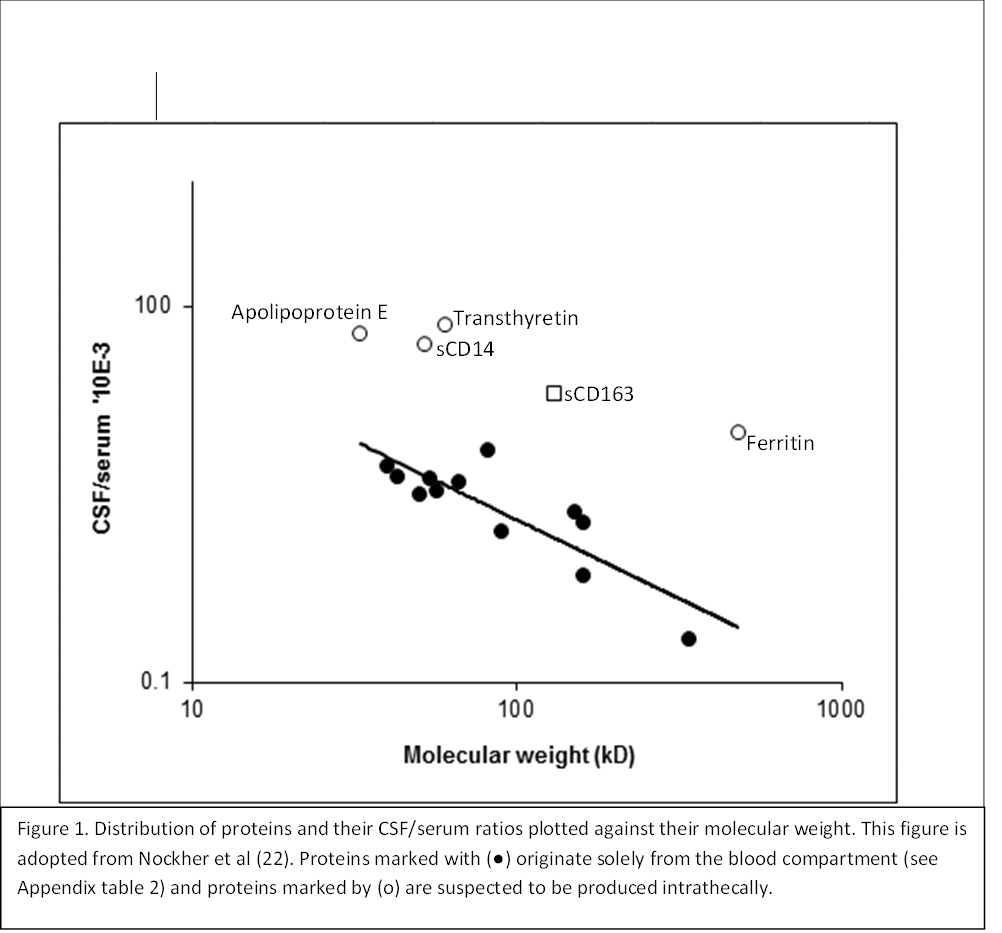


Figure S1. Distribution of proteins and their CSF/serum ratios plotted against their molecular weight (in kD). This figure is adapted from Nockher et al (22). Proteins marked with (●) originate solely from the blood compartment (see appendix table 2 below) and proteins marked by (o) are suspected to be produced intrathecally.

**Appendix calculations:**

**Calculation of intrathecal synthesis**

The % of intrathecally produced sCD163 was calculated as follows (14):

The sCD163 produced intrathecally was calculated by this formula: serum sCD163 x (CSF/serum albumin ratio) subtracted from the absolute CSF concentration. The result is presented as a percentage of the absolute CSF concentration.

**Calculation of ratio and index**

All ratios (i.e.sCD163 and albumin ratio) were derived by simply dividing the CSF concentration by the serum concentration.

The sCD163 index was calculated by dividing sCD163 ratio by albumin ratio.

**Results from the regression analyses in STATA:**

Analyses are performed on log-transformed data (i.e. lSerum). This type of analysis is described thoroughly in the STATA journal [23]. Groups in the regression analyses: 1=RRMS; 2=PPMS; 2=CIS; 4=SPMS.

**Table S3**


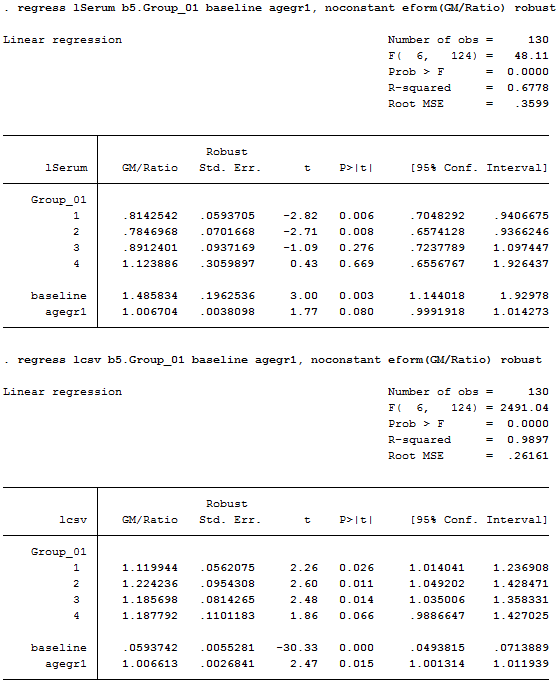


Table S3 shows the output of our regression analysis on log transformed

sCD163 serum values (lSerum)

**Table S4**


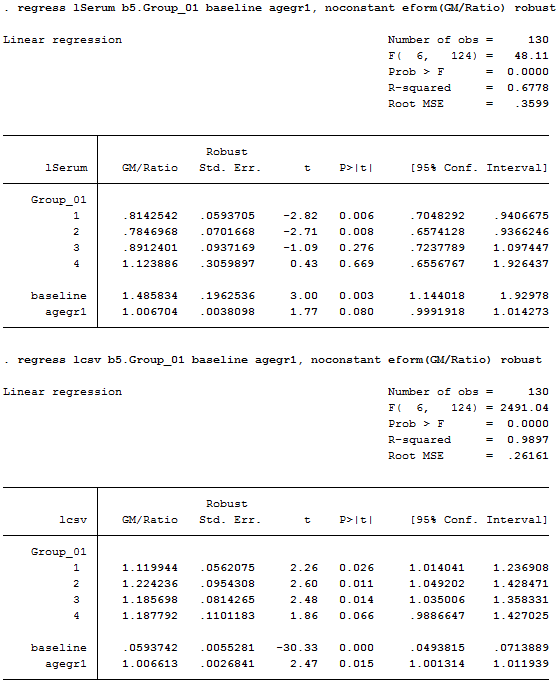


Table S shows the output of our regression analysis on log transformed

sCD163 CSF values (lcsv)

**Table S5**


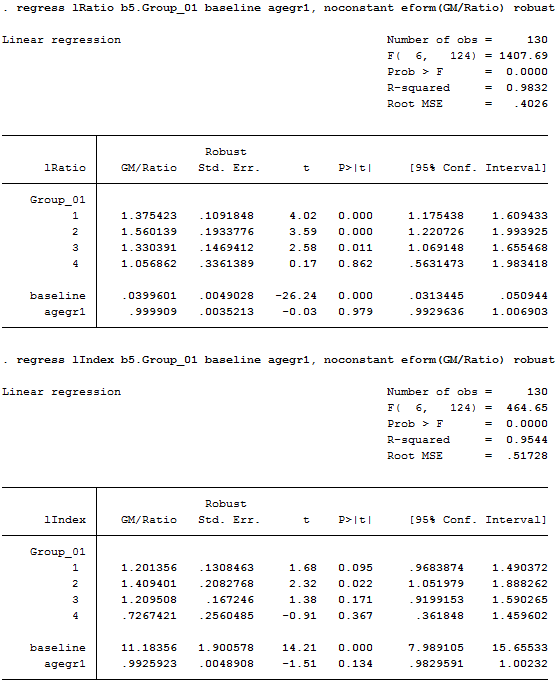


Table S5 shows the output of our regression analysis on log transformed

scd163 CSF/serum values (lRatio)

**Table S6**


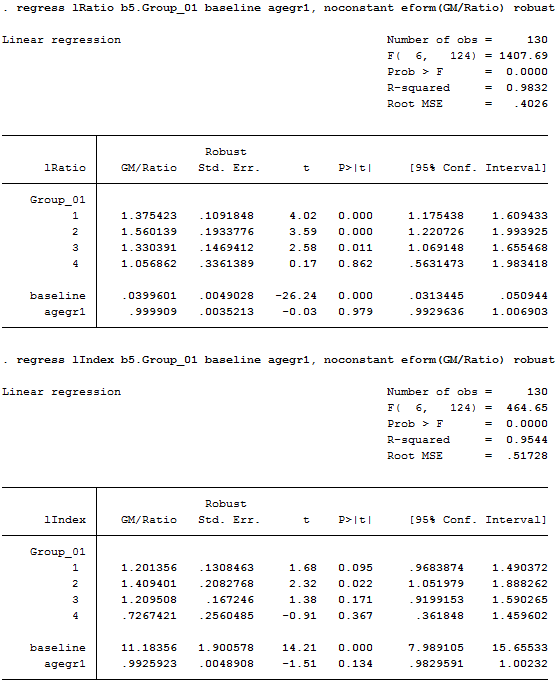


Table S3 shows the output of our regression analysis on log transformed

sCD163 index values (lIndex)

All references in Data S1 are given in the article.

For further information please contact [mortenleifms@gmail.com](mailto:mortenleifms@gmail.com)
